# ASSESSMENT OF SUBJECTIVE SEXUAL EXPERIENCES OF OLDER ADULTS

**DOI:** 10.1093/geroni/igae098.2862

**Published:** 2024-12-31

**Authors:** Janie Steckenrider

**Affiliations:** Loyola Marymount University, Los Angeles, California, United States

## Abstract

There is extensive research focusing on sexual dysfunction, decreased frequency, and sexual effects of aging, however few studies explore older adults’ subjective sexual experiences. Consequently, ageist myths prevail that older adults are neither sexually active nor desirous and the quality of their sexual activity must be extremely poor due to dysfunction and older bodies. The aim of this study was to determine how older adults assess their sexual experiences, what aspects are important, and their sexual outlook through survey research with a sample of individuals age 60+ (N=556). The results found older adults are overwhelmingly positive about their sexual experiences along with a realistic recognition of aging effects. Almost all, 85%, are sexually active, partnered and/or solo sex. Satisfaction is high indicated by 45% extremely/very satisfied with both quality and frequency and by a majority rating recent sexual experiences above average on a 1 to 10 scale and a third ranking it as “great sex” (8+). Their positivity is further evidenced as 80% describe their recent sexual experiences as fun and 67% answered exciting, despite 60% also recognizing it is predictable and more challenging, and 72% admitting it’s not their best sex ever. Sexual desire remains high as the majority look forward to their next sexual experience and 70% anticipate an unlimited number of orgasms in their future. Consistent trends were found across all 4 age groups. This study’s findings are significant for reframing the myths and demonstrating that older adults are sexually interested and having overwhelmingly positive sexual experiences.

